# Functionalized superparamagnetic iron oxide nanoparticles provide highly efficient iron-labeling in macrophages for magnetic resonance–based detection *in vivo*

**DOI:** 10.1016/j.jcyt.2017.01.003

**Published:** 2017-04

**Authors:** Jack Sharkey, Philip J. Starkey Lewis, Michael Barrow, Salamah M. Alwahsh, June Noble, Eilidh Livingstone, Ross J. Lennen, Maurits A. Jansen, Jaime Garcia Carrion, Neill Liptrott, Shareen Forbes, Dave J. Adams, Amy E. Chadwick, Stuart J. Forbes, Patricia Murray, Matthew J. Rosseinsky, Christopher E. Goldring, B. Kevin Park

**Affiliations:** 1 Department of Cellular and Molecular Physiology, Institute of Translational Medicine, University of Liverpool, Liverpool, United Kingdom; 2 UK Regenerative Medicine Platform Safety and Efficacy Hub, United Kingdom; 3 MRC Centre for Regenerative Medicine, Little France Drive, University of Edinburgh, Edinburgh, United Kingdom; 4 Department of Chemistry, University of Liverpool, Liverpool, United Kingdom; 5 Cardiovascular Sciences, Queens Medical Research Institute, University of Edinburgh, Edinburgh, United Kingdom; 6 Edinburgh Preclinical Imaging, University of Edinburgh, Edinburgh, United Kingdom; 7 MRC Centre for Drug Safety Science, Ashton Street, University of Liverpool, Liverpool, United Kingdom; 8 European Nanomedicine Characterisation Laboratory (EU-NCL), Department of Molecular and Clinical Pharmacology, University of Liverpool, Liverpool, United Kingdom

**Keywords:** cell therapy, cell tracking, liver fibrosis, macrophage, MRI

## Abstract

**Background aims:**

Tracking cells during regenerative cytotherapy is crucial for monitoring their safety and efficacy. Macrophages are an emerging cell-based regenerative therapy for liver disease and can be readily labeled for medical imaging. A reliable, clinically applicable cell-tracking agent would be a powerful tool to study cell biodistribution.

**Methods:**

Using a recently described chemical design, we set out to functionalize, optimize and characterize a new set of superparamagnetic iron oxide nanoparticles (SPIONs) to efficiently label macrophages for magnetic resonance imaging–based cell tracking *in vivo*.

**Results:**

A series of cell health and iron uptake assays determined that positively charged SPIONs (+16.8 mV) could safely label macrophages more efficiently than the formerly approved ferumoxide (−6.7 mV; Endorem) and at least 10 times more efficiently than the clinically approved SPION ferumoxytol (−24.2 mV; Rienso). An optimal labeling time of 4 h at 25 µg/mL was demonstrated to label macrophages of mouse and human origin without any adverse effects on cell viability whilst providing substantial iron uptake (>5 pg Fe/cell) that was retained for 7 days *in vitro*. SPION labeling caused no significant reduction in phagocytic activity and a shift toward a reversible M1-like phenotype in bone marrow–derived macrophages (BMDMs). Finally, we show that SPION-labeled BMDMs delivered *via* the hepatic portal vein to mice are localized in the hepatic parenchyma resulting in a 50% drop in T2* in the liver. Engraftment of exogenous cells was confirmed via immunohistochemistry up to 3 weeks posttransplantation.

**Discussion:**

A positively charged dextran-coated SPION is a promising tool to noninvasively track hepatic macrophage localization for therapeutic monitoring.

## Graphical Abstract

**Figure:**
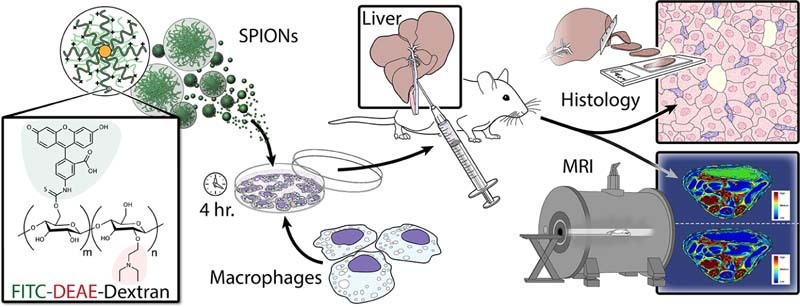

## Introduction

Cell-based therapy offers an exciting new approach in the field of regenerative medicine to treat a range of diseases. Tracking cells after transplantation through medical imaging is considered an indispensable tool to ensure appropriate cell localization, migration and engraftment in the host tissue. There is a pressing need to develop new techniques to noninvasively detect and track transplanted cells *in vivo* using sensitive and safe tracking agents. Superparamagnetic iron oxide nanoparticles (SPIONs) are a versatile class of magnetic resonance imaging (MRI)-based contrast agents that have been used clinically to detecthepatocellular carcinomas in patients [1], [2] and have potential as magnetic fluid hyperthermia treatment for cancers as well as magnetic targeting of drugs [3]. More recently, several reports have successfully employed SPIONs as a means to label cells *in vitro* (primarily macrophages, which readily ingest SPIONs) before transplantation and subsequently track cells via MRI [4], [5]. Obtaining sufficient iron uptake in cells is a challenge and the limiting factor for MRI sensitivity. To improve uptake, several approaches have been used previously, including post-modification steps [6] or use of transfection agents [7], which can elicit toxicity [8]. In 2009, two clinically approved SPIONs were removed from the market for commercial reasons, ferumoxide (Endorem, Guerbet) and ferucarbotran (Resovist, Schering). Ferumoxytol (Rienso, Takeda/AMAG Pharmaceuticals) is still available clinically for the treatment of anemia in parts of Asia and the United States, but not in Europe. Therefore, there is currently a lack of suitable MRI-based contrast agents that can be used in both the laboratory and the clinic for cell-tracking purposes.

Our group recently described a technique to synthesize SPIONs with a modified dextran coating that contains diethylaminoethyl (DEAE) and fluorescein isothiocyanate (FITC) moieties that confer a positive charge and green fluorescent properties (GFPs) to the nanoparticles, respectively [9]. Chemically and structurally, the novel SPIONs are comparable to the formerly clinically approved SPIONs in having a dextran-coating and a similar sized iron core of approximately 60 nm. Here, we have used these novel SPIONs to label macrophages of murine and human origin. Macrophages represent a promising cell therapy for the treatment of liver fibrosis by reducing fibrotic scarring and improving liver regeneration and function through several mechanisms, including the expression of metalloproteinase enzymes, which degrade fibrotic scars, clearance of cellular debris and increased expression of cytokines implicated in the regenerative processes of the liver such as vascular epithelial growth factor [10], [11]. Phase I/II clinical trials are currently in progress (Macrophage Therapy for Cirrhosis [MATCH] study). The availability of a suitable cell-tracking agent for macrophage therapy would potentially complement the design of prospective clinical trials and support clinical use by monitoring appropriate localization and engraftment of cells in the host.

Therefore, to assess whether the SPIONs are suitable MRI-based contrast agents for macrophage therapy, we have performed a series of investigations to examine the effect of SPION-labeling on these cells. Primary macrophages were differentiated *in vitro* from mouse bone marrow (bone marrow–derived macrophages [BMDMs]) or from human monocytes (monocyte-derived macrophages [hMDMs]) using defined protocols. Both sources provide highly enriched populations of mature and functionally active macrophages as previously reported [11], [12]. Our group has recently described a method to control the synthetic approach to change the size and electrostatic charge of dextran-coated SPIONs [9]. Here we have investigated how modulation of these physicochemical properties at the particle level can influence the suitability of these molecules as macrophage contrast agents. Hence, macrophages were labeled with either novel functionalized SPIONs, previously clinically approved, or clinically approved SPIONs that served as comparators. Safety studies (cell viability/cytotoxicity) and iron uptake experiments were performed with respect to dose and time to identify the most appropriate labeling protocol in these cells. Due to the plasticity of macrophages, we further sought to test whether iron labeling caused any change in cell phenotype or function. Finally, we performed a longitudinal MRI study to monitor the biodistribution of SPION-labeled BMDMs *in vivo* after transplantation. In this way, we have been able to show that macrophages localize into the liver parenchyma after transplantation, which can be monitored for several weeks. We have thus developed a SPION that can efficiently label macrophages, is biocompatible and can serve as a suitable tracking agent to noninvasively monitor hepatic localization of macrophages *in vivo*.

## Methods

### BMDMs

Femurs and tibias of C57/BL6 male mice (8–10 weeks old) were collected in Hank's Balanced Salt Solution (Gibco) containing penicillin/streptomycin. In a sterile fume hood, muscle tissues were removed. Bone marrow was flushed out with Dulbecco's Modified Eagle's Medium (DMEM):F12 (1:1) cell culture media (Gibco) supplemented with 10% fetal bovine serum (v/v), 1 × glutamine and 1 × penicillin/streptomycin. Mouse bone marrow was centrifuged (400*g*, 5 min) and then resuspended in fresh supplemented DMEM:F12 media (20 mL) containing 20 ng/mL murine recombinant macrophage colony-stimulating factor (mCSF; Peprotech). Bone marrow suspensions were then transferred to sterile ultra-low attachment flasks (Corning) and incubated at 37°C, 5% CO_2_. Every second day, 10% of the media was replaced with fresh media containing 400 ng mCSF. After 7 days, mature macrophages were fully differentiated (as determined by fluorescence-activated cell sorting [FACS]/morphological assessment) and subsequently used for iron-labeling experiments. In some experiments, BMDMs were primed toward an M1- or M2-like phenotype by the overnight addition of recombinant murine interferon-γ (PeproTech) and lipopolysaccharides (lipopolysaccharides from *Escherichia coli*; Sigma) or recombinant murine interleukin (IL)-4 (PeproTech; M1 and M2, respectively).

### hMDMs

CD14^+^ monocytes were harvested via apheresis from healthy volunteers as described in Moore *et al.* [12]. Monocytes were frozen in liquid nitrogen in cryopreservation solution. For SPION-labeling experiments, high-density monocyte aliquots were defrosted in a water bath, washed, and resuspended in Iscove's Modified Eagle Medium supplemented with 10% fetal bovine serum and 100 ng/mL human recombinant mCSF (Miltenyi) in low-attachment flasks (Corning) at 2 × 10^6^ monocytes/mL for 7 days, replacing mCSF every second day. After 7 days, hMDMs were harvested as described, counted and incubated overnight in 96-well plates (4 × 10^4^/well) before viability studies were performed.

### SPION labeling of macrophages

SPIONs were synthesized as described in supplementary methods. Ferumoxytol (Rienso, Takeda Pharmaceuticals) was sourced from Takeda Pharmaceuticals [13]. SPIONs were diluted in fresh supplemented media (as described) containing 1% fetal bovine serum (v/v) and immediately applied to cells at the appropriate doses (0–100 µg Fe/mL) and times (0–168 h) in microplates. Cell health assessments were performed independently by two investigators to ensure reproducibility.

### Cell viability and cytotoxicity analysis

Cell viability and cytotoxicity was assessed by measurement of cellular adenosine triphosphate (ATP) content using CellTiter-Glo Luminescent Cell Viability Assay (Promega) and the Cytotoxicity Detection Kit based on lactate dehydrogenase leakage (LDH) (Roche) according to the manufacturer's instructions.

### Iron uptake quantification (ferrozine assay)

Cellular iron levels were determined using an assay based on previously reported work [14], [15]. Briefly, cells were lysed in 1.2 mol/L HCl (50 µL) and detached from the plate surface using a pipette tip. Acidified cell lysates were transferred into microtubes and digested at 65°C for 2 h. Lysates were allowed to cool and then diluted with dH20 (1:1). Ferrozine reagent (30 µL–5 mol/L ammonium acetate, 2 mol/L ascorbic acid, 6.5 mmol/L 3-(2-Pyridyl)-5,6-diphenyl-1,2,4-triazine, 15 mmol/L neocuproine) was added to each sample, vortexed and allowed to equilibrate for 30 min to allow color development. Absorbance was measured at 570 nm on a microplate reader (Fluostar Omega, BMG Labtech). A standard curve was used using known iron standards (Sigma Aldrich), and samples were interpolated from the standard curve to quantify iron content per cell.

### Prussian blue staining

Cellular iron content was visualized using the Prussian blue stain as outlined in supplementary methods.

### Gene expression analysis

The expression of a number of mRNAs chosen for their role in macrophage polarization were quantified using quantitative polymerase chain reaction. The following primers messenger RNAs were detected: IL-1b, IL-12b, IL-10, tumor necrosis factor (TNF)-α, CD206 (MRC), Arg-1, Chi3L3 (QuantiTect Primer Assay, Qiagen). Each sample was measured in triplicate and normalized to glyceraldehyde-3-phosphate dehydrogenase.

### Flow cytometry

To assess the phagocytic ability of BMDMs incubated with SPIONs, BMDMs were incubated with fluorescently labeled *Saccharomyces cerevisiae* (Zymosan A *S. cerevisiae* BioParticles, Texas Red conjugated, ThermoFisher Scientific). Flow cytometry was also used to assess the cell surface markers CD86 and CD206 on BMDMs.

### Transplantation and MRI

For cell-tracking studies, BMDMs were differentiated as described from 10-week-old male mice that constitutively express activated enhanced GFP (aEGFP) under a phosphoglycerate kinase (PGK) promoter on a C57BL6/J background (see Gilchrist *et al.*) [16]. BMDMs were counted and incubated with SPION (DEAE-Dex 1:4) for 4 h at 25 µg Fe/mL in low-attachment flasks. SPION-labeled BMDMs (1 × 10^6^) were transplanted via the hepatic portal vein (HPV) of healthy wild-type C57BL6/J male mice. The HPV was accessed via midline laparotomy using aseptic technique. Anesthesia was induced and maintained with gaseous oxygen/isofluorane. After injection, the peritoneal wall and skin were sutured. Buprenorphine (0.05 mg/kg, subcutaneous) was given for analgesia with saline (500 µL, subcutaneous). Experimental animal protocols were performed in accordance with the approved guidelines under a license granted under the Animals (Scientific Procedures) Act 1986 and approved by the University of Edinburgh Animal Ethics Committee.

MRI was performed the day before transplantation (*n* = 4, baseline measurements). The same mice were scanned again on days 6 (*n* = 4), 13 (*n* = 3), 20 (*n* = 2) and 27 (*n* = 1) post-transplantation to monitor biodistribution over 4 weeks, the timeframe through which macrophages have been described to provide therapeutic action. One mouse was culled per time point for histological assessment. Anesthetized mice were scanned on a 7T Agilent MRI using a 33-mm inner diameter radiofrequency coil for signal transmission and reception. For T2* mapping, images were acquired in axial orientation using a respiratory-gated gradient echo sequence with the following parameters: repetition time 60 ms; echo time 1, 2, 3, 5, 7, 9, 12 and 15 ms, matrix size 128 × 128, field of view 35.0 × 35.0 mm, flip angle 15°, slice thickness 2 mm and two signal averages. Axial sections were taken from the upper abdomen (to capture mainly liver) and lower abdomen (to capture several abdominal organs). Three regions of interest (ROIs) were taken from each organ to quantify an average mean. T2* maps were generated and quantified in FIJI [17] using the MRI Analysis Calculator plug-in.

### Immunohistochemistry

Detection of EGFP was performed on unfixed frozen sections. Briefly, 7-µm sections were cut on a cryostat and collected on SuperFrost Plus slides and fixed according to Jockusch *et al*., using formaldehyde vapor fixation [18]. Sections were washed for 5 min with phosphate buffered saline, and blocked with a protein block (Spring Bioscience) for 30 min at room temperature. Sections were then incubated with polyclonal goat anti-GFP (1:200) for 60 min at room temperature, extensively washed and then incubated with donkey anti-goat Alexa 488 (1:300, Life Technologies) for 1 h in a dark environment. Slides were mounted with 4′,6-diamidino-2-phenylindole (DAPI)-fluoromount G, and were examined under a fluorescence microscope (Nikon Eclipse E600). Negative controls were obtained by omission of the primary antibody or using isotype goat immunoglobulin G (1:200) instead of the primary antibody. Cryosections from aEGFP liver served as a positive control.

### Statistical methods

Data points are presented as the mean value around the standard deviation, unless otherwise stated. Statistical analysis was performed in GraphPad Prism 6 (Graphpad Software). Data sets were tested for normality using the Shapiro-Wilk test. For tests containing two groups, a Student's *t*-test was performed; a one-way analysis of variance was performed for tests containing more than two groups.

### Additional methods

Additional and more detailed methods are provided in supplementary materials.

## Results

### Clinically approved SPIONs are biocompatible with BMDMs

**Figure 1:**
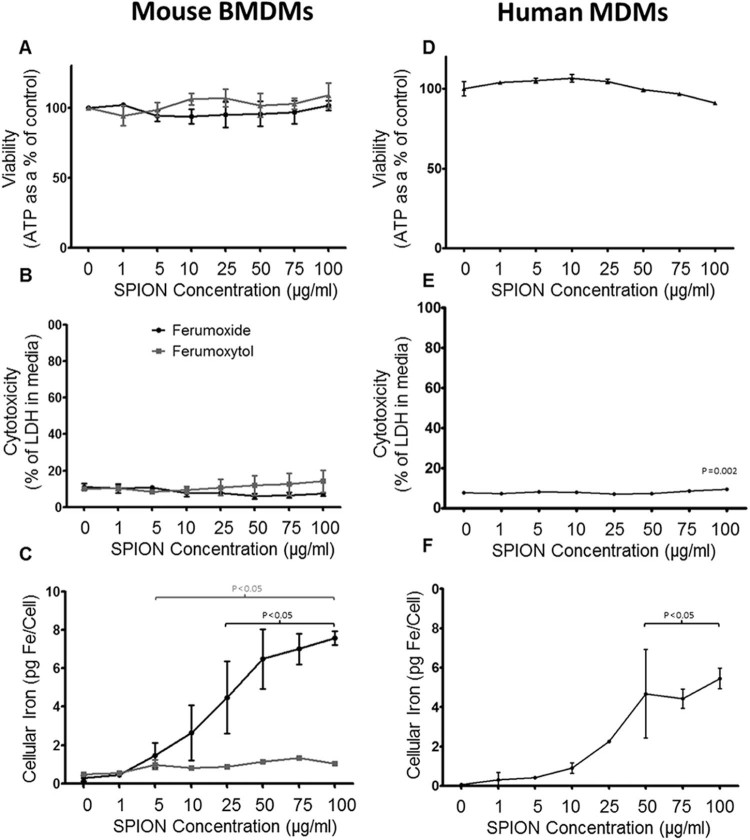
Biocompatibility of clinically approved SPIONs. Cell health (A and D), cytotoxicity (B and E) and intracellular iron content (C and F) assays in murine BMDMs incubated with three commercial SPIONs for 24 h or human MDMs incubated with ferumoxide only. Macrophages were incubated with either ferumoxide (black lines) or ferumoxytol (gray lines) as indicated in the panels. Bars show the mean value per group around the standard deviation (error bars). Statistical significance (*P*) to respective controls (0 µg/mL SPION) was determined by a one-way analysis of variance and indicated by *P* values in corresponding colors to the lines.

Two clinically approved SPIONs (ferumoxide and ferumoxytol) were incubated with murine BMDMs to assess their effect on cell health and the degree of uptake by BMDMs. BMDMs were incubated with ferumoxide or ferumoxytol over a range of concentrations (0–100 µg/mL) for 24 h. None of the commercial SPIONs caused any significant reduction in cell viability as assessed by ATP activity (Figure 1A) or significant cell death as assessed by LDH release (Figure 1B). BMDMs incubated with ferumoxytol had a peak iron content of 1.3 pg Fe/cell whereas ferumoxide had peak iron contents of 7.6 pg Fe/cell respectively (Figure 1C).

### Biocompatibility and iron uptake of dextran-coated SPIONs depends on surface charge

**Figure 2:**
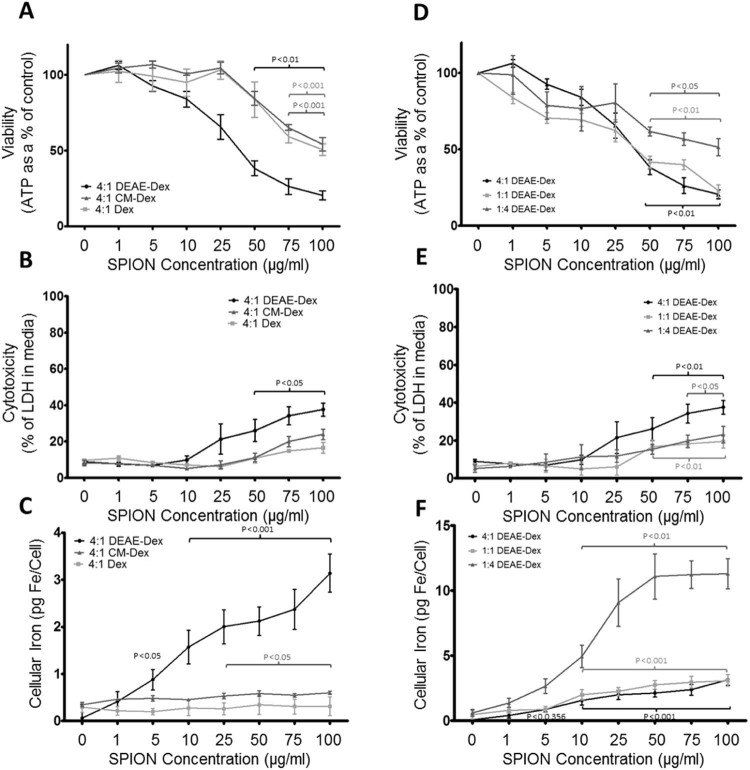
Biocompatibility of SPIONs is dependent on surface charge and iron core size. Cell health (A and D), cytotoxicity (B and E) and intracellular ion content (C and F) of BMDMs incubated with dextran coated SPIONs with varying surface charges (A–C) or a positive surface charge and varying iron core sizes (D–F). The positively charged DEAE-Dex 4:1 (A–C; black lines), the neutral 4:1 Dex (A–C; light gray lines) and the negatively charged CM-Dex 4:1 (A–C; gray lines) were incubated with BMDMs. The DEAE-Dex 4:1 (D–F; black lines) has a hydrodynamic diameter of 39.7 nm, the 1:1 Dex (D–F; light gray lines) 47.3 nm and the DEAE-Dex 1:4 (D–F; gray lines) 68 nm. Statistical significance (*P*) to respective controls (0 µg/mL SPION) was determined by a one-way analysis of variance and indicated by *P* value in corresponding color to lines.

To determine the optimal parameters required for iron labeling in macrophages, we designed, synthesized and tested three dextran particles as described in Table I. The three particles had a neutral surface charge (Dex), a positive surface charge (DEAE-Dex 4:1) or a negative surface charge (CM-Dex) [20] and were incubated with BMDMs for 24 h over a range of concentrations (0–100 µg/mL). Dex and CM-Dex caused significant reductions in cell viability at 75 µg/mL (59% and 65%, respectively) and 100 µg/mL (50% and 54%, respectively) as determined by the ATP assay. DEAE-Dex 4:1 ratio caused significant reductions in cell viability at 50, 75 and 100 µg/mL (38%, 26% and 20% respectively) (Figure 2A). Dex and CM-Dex caused no significant increases in cell death as determined by the LDH assay (Figure 2B), whereas DEAE-Dex 4:1 caused significant increases in cell death at 75 and 100 µg/mL (34% and 37% respectively). However, Dex and CM-Dex only modestly labeled BMDMs providing peak cellular iron contents of 0.3 and 0.6 pg Fe/cell respectively, whereas DEAE-Dex 4:1 caused a substantial dose-dependent increase in iron content, which reached 3.1 pg Fe/cell (Figure 2C).

### Biocompatibility and iron uptake of dextran-coated SPIONs depends on core size

Because the positively charged dextran-coated SPIONs (DEAE-Dex 4:1) provided the best uptake, we next altered the core size of the positively charged SPION to further improve labeling efficiency in BMDMs (Table I). Three SPIONs were synthesized with increasing hydrodynamic diameter using different ratios of DEAE and dextran (DEAE-Dex 4:1, DEAE-Dex 1:1 and DEAE-Dex 1:4) [19]. These candidates were incubated with BMDMs for 24 h. DEAE-Dex 1:1 and DEAE-Dex 1:4 caused significant reductions in cell viability at 50 (42% and 62%, respectively), 75 (40% and 56%, respectively) and 100 µg/mL (23% and 51%, respectively) as determined by the ATP assay (Figure 2D). However, neither DEAE-Dex 1:1 nor DEAE-Dex 4:1 caused any significant increase in cell death as determined by the LDH assay (Figure 2E). DEAE-Dex 1:1 led to intracellular iron levels that reached 3.1 pg Fe/cell, and DEAE-Dex 1:4 led to a dose-dependent increase in intracellular iron levels that reached 11.3 pg Fe/cell (Figure 2F).

### Reducing labeling time of DEAE-Dex 1:4 improves biocompatibility and labels macrophages at sufficient iron levels required for MRI

**Figure 3:**
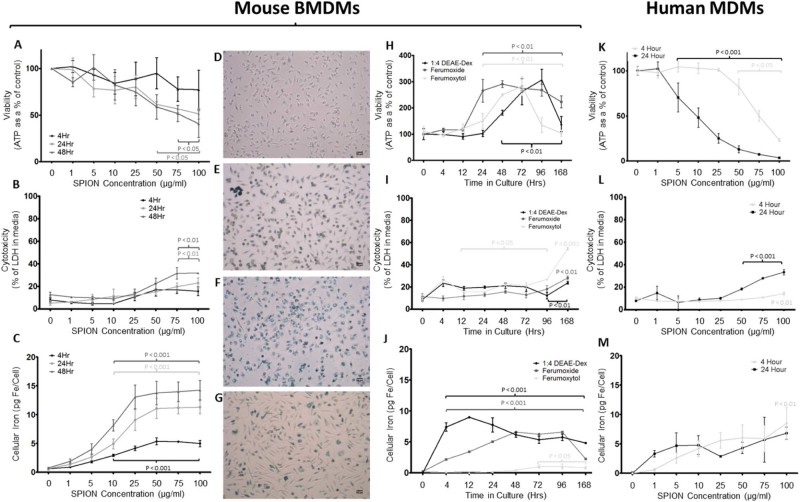
Biocompatibility of SPIONs is dependent on labeling time in murine and human macrophages. Cell health (A), cytotoxicity (B) and intracellular ion content (C) of BMDMs incubated with DEAE-Dex 1:4 for either 4 h (black lines), 24 h (light gray lines) or 48 h (gray lines). Prussian blue staining shows intracellular iron after a 4 h (D), 24 h (E) and 48 h (F) incubation and 7 days post-24 h incubation (G). A 7 day time course study was performed in BMDMs labeled for 4 h with DEAE-Dex 1:4 (black lines), ferumoxide (gray lines), or ferumoxytol (light gray lines) and then replaced with normal media to determine cell viability (H), cytotoxicity (I), and iron labeling (J). (K–M) Human MDMs were incubated with DEAE-Dex 1:4 for 4 h (black lines) or 24 h (light gray lines) to determine cell viability (K), cytotoxicity (L) and iron labeling (M). Statistical significance (*P*) to respective controls (0 µg/mL SPION) was determined by a one-way analysis of variance and indicated by *P* values in corresponding colors to lines.

We next investigated DEAE-Dex 1:4 further with varying incubation times with BMDMs, from 4 h up to 48 h, and also 7 days after a 24 h labeling period (Figure 3A–J). A 4 h incubation caused no significant decrease in cell viability or increase in cytotoxicity, resulting in a peak cellular iron content of 5.4 pg Fe/cell. A 24 h incubation with DEAE-Dex 1:4 caused significant reductions in cell viability (Figure 3A) at 50, 75 and 100 µg/mL (61%, 56% and 51% respectively) and significant increases in cell death (Figure 3B) at 75 and 100 µg/mL (19.7% and 23%, respectively). A 48 h incubation caused significant reductions in viability at 75 and 100 µg/mL (51% and 40%, respectively) and significant increases in cell death at 75 and 100 µg/mL (31.6% and 31.9%, respectively). Iron uptake (Figure 3C) showed a dose-dependent increase in both 24 h and 48 h incubations with peak cellular iron contents of 11.3 and 14.3 pg Fe/cell, respectively. Prussian blue staining of BMDMs shows intracellular iron at 4, 24, 48 and 7 days post-24 h incubation with DEAE-Dex 1:4 (Figure 3D–G).

Because a 4 h incubation of DEAE-Dex 1:4 at 25 µg/mL provided substantial iron-labeling but caused no change in any index of cell health, we proceeded to investigate cell health and iron content up to 7 days post-labeling compared with ferumoxide (at 25 µg/mL) and ferumoxytol (at 100 µg/mL to promote iron labeling). Using this labeling protocol, we observed no drop in ATP (Figure 3H) levels at any time with any of the SPIONs tested. Interestingly, we observed an approximate twofold to threefold increase in ATP levels post-labeling peaking at 72–96 h after labeling with all three SPIONs. However, we have also observed this effect in unlabeled BMDMs, and therefore there was no difference over controls (data not shown), which suggests this is a function of the natural changes in BMDM physiology during the course of *in vitro* culture.

Furthermore, we observed no increase in LDH leakage at any time point in BMDMs labeled with DEAE-Dex 1:4 in contrast to the comparator SPION, ferumoxytol, which caused a twofold increase (Figure 3I). However, the ATP data suggest that the cells are still viable at this time point (Figure 3H), and therefore the increase in LDH leakage might be due to increased metabolic activity rather than overt cytotoxicity per se. Iron labeling in BMDMs labeled with DEAE-Dex 1:4 outperformed the comparator SPIONs with cellular iron levels of 4.8 pg/cell at 7 days compared with 2.3 and 0.8 pg Fe/cell for ferumoxide and ferumoxytol, respectively (Figure 3J). BMDMs labeled with DEAE-Dex 1:4 had 46% less cellular iron at 7 days (4.8 pg Fe/cell) compared with the maximum labeling level at 12 h (8.9 pg Fe/cell), suggesting excretion of SPIONs. Nevertheless, we found this iron concentration sufficient to provide MRI contrast.

To evaluate the biocompatibility of DEAE-Dex 1:4 in hMDMs, we incubated this SPION for 4 and 24 h (Figure 1, Figure 3). Similar to murine BMDMs, there was a time- and dose-dependent toxicity evident. ATP levels dropped 18%, 50% and 76% at 50, 75 and 100 µg/mL, respectively (Figure 3K). This effect was pronounced at 24 h with ATP levels dropping 87%, 92.6% and 96% at the same concentrations. Cytotoxicity was also evidenced through LDH release which increased 1.4-fold at 100 µg Fe/mL after 4 h incubation but 4.3-fold after 24 h incubation at the same concentration (Figure 3L). However, iron labeling was more variable between wells in hMDMs, probably due to toxicity being a confounding factor in these cells, which were more sensitive to SPION treatment. In hMDMs, we observed an iron content of 5.6 pg Fe/cell at 25 µg/mL after 4 h (Figure 3M). An iron content of 2.9 pg Fe/cell was measured after 24 h labeling at the equivalent SPION concentration suggesting cell death was reducing the average measurement of iron across the population. Therefore, a reduced labeling time of 4 h at 25 µg/mL provides efficient iron labeling that is well tolerated in murine and human macrophages and is well retained for 7 days *in vitro*.

### The effect of incubation with DEAE-Dex 1:4 on BMDM function and phenotype

**Figure 4:**
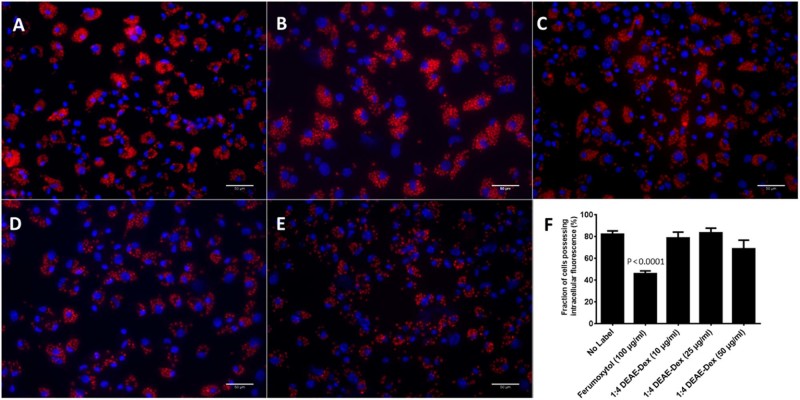
Immunofluorescence images showing differently treated BMDMs incubated with *S. cerevisae* particles conjugated to Texas red (red) for 2 h and subsequently stained with DAPI (blue). Extracellular fluorescence was quenched with 0.4% trypan blue. BMDMs were either not treated (A), treated with 100 µg/mL ferumoxytol (B), 10 µg/mL DEAE-Dex 1:4 (C), 25 µg/mL DEAE-Dex 1:4 (D) or 50 µg/mL DEAE-Dex 1:4 (E) for 24 h before staining. The aforementioned treated cells were also analyzed using FACS to detect the fraction of cells that possessed intracellular fluorescence (F). Statistical significance (*P*) to respective controls (0 µg/mL SPION) was determined by a one-way analysis of variance and indicated by *P* values in corresponding colors to lines.

To determine whether DEAE-Dex 1:4 incubation and subsequent uptake by BMDMs affected their function, the phagocytic ability was assessed. BMDMs were incubated with dextran-coated SPIONs and ferumoxytol for 24 h followed by incubation with fluorescently labeled *S. cerevisiae* and assessed by either FACS or microscopy. Incubation with 10, 25 or 50 µg/mL DEAE-Dex 4:1 caused no significant reduction in the fraction of fluorescently labeled cells as assessed by FACS (Figure 4F). However, incubation with 100 µg/mL ferumoxytol caused significant reductions in the fraction of labeled cells (45.6%). Microscopy images also show that ferumoxytol (Figure 4B) leads to fewer yeast containing cells.

**Figure 5:**
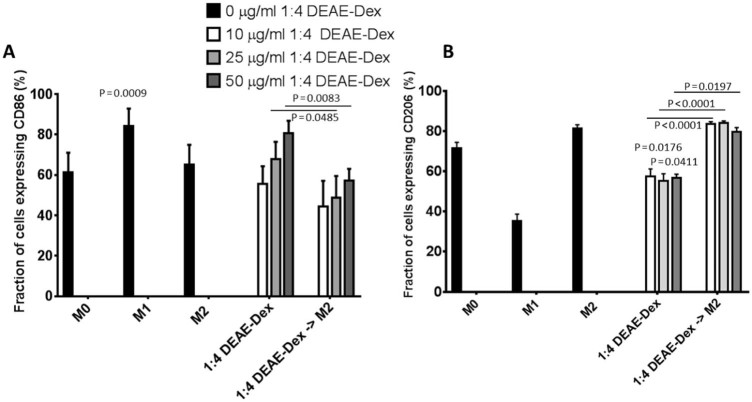
FACS analysis of CD86 (A) and CD206 (B) in differently treated BMDMs. BMDMs were either treated for 24 h with media containing MCSF-1 only (control macrophages), primed toward an M1-like phenotype, primed toward an M2-like phenotype, incubated with DEAE-Dex 1:4 alone or DEAE-Dex 1:4 followed by priming toward an M2-like phenotype for 24 h. BMDMs were either not labeled with DEAE-Dex 1:4 (black bars), or labeled with DEAE-Dex 1:4 at concentrations of 10 µg/mL (white bars), 25 µg/mL (light gray bars) or 50 µg/mL (dark gray bars). Statistical significance (*P*) was determined by a one-way ANOVA and indicated by *P* values. *P* values compared with M0 macrophages.

After this, the effect of DEAE-Dex 1:4 on BMDM phenotype was investigated by assessing the expression of the cell surface markers, CD86 and CD206, and also by quantifying the expression of a number of mRNAs which are indicative of an M1-like (IL-1b, IL-12b, TNFα) or M2-like (CD206, IL-10, Arg-1, Chi3l3) phenotype. The expression of CD86 increased in a dose-dependent manner when incubated with 10, 25 or 50 µg/mL DEAE-Dex 1:4. This increase in CD86 expression could be significantly reduced to 48.9% and 57.2% at 25 and 50 µg/mL, respectively, if BMDMs were incubated with IL-4 for 24 h post-labeling with DEAE-Dex 1:4 (Figure 5A). The expression of CD206 was decreased in response to 10, 25 and 50 µg/mL DEAE-Dex 1:4 (Figure 5B). However, this decrease could be reversed by incubation with IL-4 after DEAE-Dex labeling to 83.6%, 84.1% and 79.7% for 10, 25 and 50 µg/mL DEAE-Dex 1:4.

**Figure 6:**
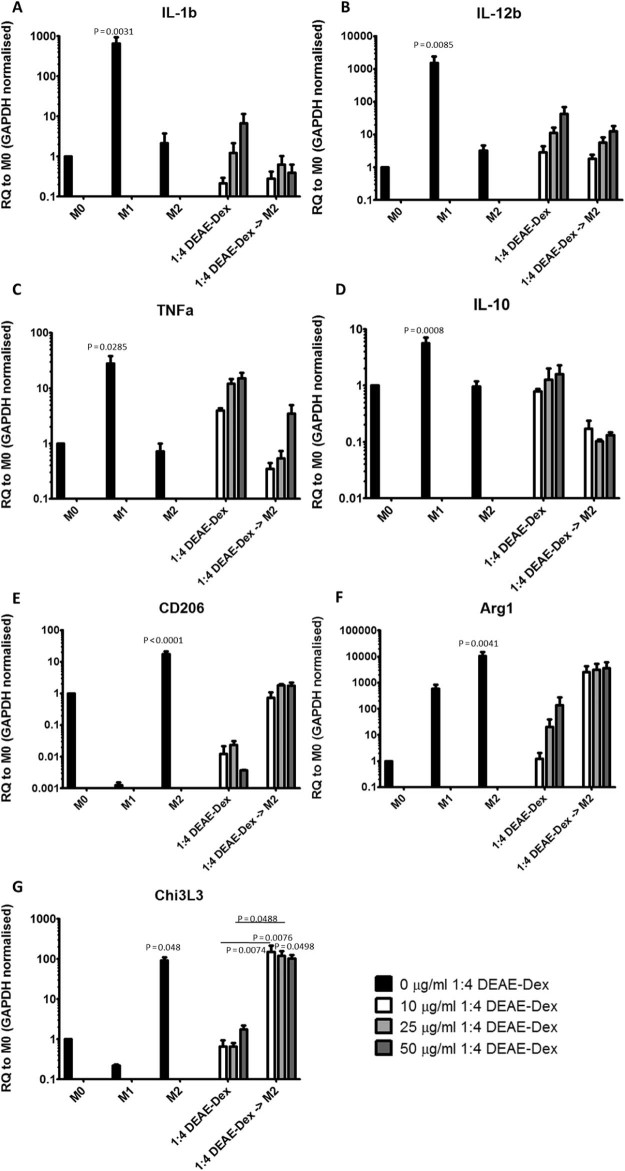
mRNA expression of genes that may suggest an M1- or M2-like phenotype in BMDMs treated with various conditions. The expression of IL-1b (A), IL-12b (B), IL-10 (C), TNF-α (D), CD206 (E), Arg-1 (F) and Chi3L3 (G) were quantified. BMDMs were either treated for 24 h with media containing MCSF-1 only (control macrophages), primed toward an M1 like phenotype, primed toward an M2-like phenotype, incubated with DEAE-Dex 1:4 alone or DEAE-Dex 1:4 followed by priming toward an M2 like phenotype for 24 h. BMDMs were either not labeled with DEAE-Dex 1:4 (black bars), or labeled with DEAE-Dex 1:4 at concentrations of 10 µg/mL (white bars), 25 µg/mL (light gray bars) or 50 µg/mL (dark gray bars). Statistical significance (*P*) was determined by a one-way analysis of variance and indicated by *P* values. *P* values compared to M0 macrophages.

mRNA expression of the M1-like phenotypic markers IL-1b, IL-12b and TNFα were significantly increased post-incubation with lipopolysaccharides and interferon-γ and increased when incubated with 10, 25 or 50 µg/mL DEAE-Dex 4:1. Expression of IL-1b, IL-12b and TNFα was decreased if BMDMs were incubated with IL-4 post-labeling with DEAE-Dex 1:4 (Figure 6A,B,D). The mRNA expression of the M2-like markers IL-10, CD206, Arg1 and Chi3L3 were significantly elevated post incubation with IL-4. The expression of the CD206 and Arg1 were decreased post-incubation with DEAE-Dex 1:4, whereas IL-10 and Chi3L3 remained relatively unchanged. The decreases in the expression of CD206 and Arg-1 were reversed by post-incubation with IL-4 for 24 h post-labeling with DEAE-Dex 1:4 (Figure 6C,E,F).

### Noninvasive MRI-based detection of SPION-labeled BMDMs *in vivo*

To test whether SPION-labeled BMDMs could be detected *in vivo* via MRI, we designed a biodistribution study. We labeled BMDMs using a protocol (25 µg Fe/mL, 4 h) that was well-tolerated and provided substantial iron loading. BMDMs were differentiated from bone marrow harvested from double homozygous aEGFP-97 mice that express EGFP constitutively and ubiquitously, including in macrophages [16]. FACS analysis showed that BMDMs (CD45^+^, CD11b, F4/80^+^ve) from aEGFP mice were 98% GFP-positive (supplementary Figure S4). SPION-labeled EGFP-positive BMDMs were transplanted (1 × 10^6^) to WT mice via the hepatic portal vein to mirror the number of cells and dosing route required for liver fibrosis therapy [11]. Mice were scanned before (baseline) and after (at 6, 13, 20 and 27 days) transplantation. The residual injectate was retained and placed back in culture for 48 h. Immunocytochemistry and Prussian blue staining confirmed that donor cells were viable, expressed GFP and were iron-rich as expected (supplementary Figure S4A,B).

**Figure 7:**
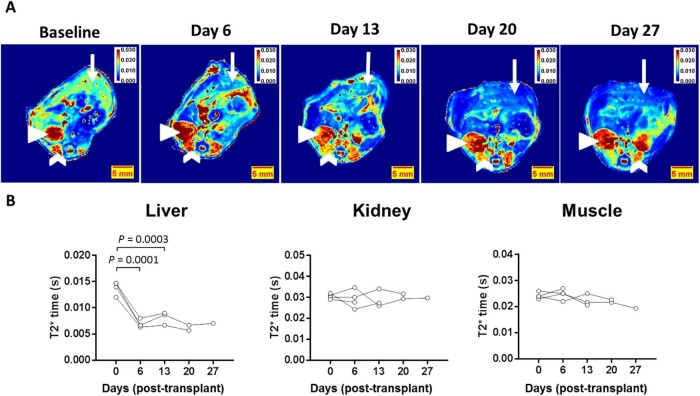
MRI detection of SPION-labeled macrophages indicates hepatic localization. (A) Axial T2* maps of the abdomen were generated from eight echo times in mice before (baseline, day 0) and after (days 6, 13, 20 and 27) transplantation of 1 × 10^6^ SPION-labeled BMDMs into the hepatic portal vein. False-color maps show representative images per time point. Mice were scanned in the dorsal position (spinal column visible at the bottom of maps). The liver is positioned at the top of the scan (white arrows) with vasculature visible. Kidneys are marked with the white arrowhead, and muscle tissue surrounding the spinal cord is marked with the white chevron. A drop in liver T2* relaxation is characterized in the maps by a switch from green (baseline) to blue (post transplantation). (B) Three ROIs were taken from each organ to quantify an average mean from pixel analysis on an animal per animal basis. Each organ is represented in the panels showing a liver-specific drop in T2* time indicative of SPION-labeled BMDM localization. Consecutive MRI scans from the same mice are connected by black line. *P* values are provided from statistical analysis where n ≥ 3, one-way analysis of variance.

MRI maps from axial sections of the abdomen in T2* scanning format showed an average 50% drop in liver T2* time after 6 days (versus baseline), indicative of hepatic localisation of SPION-labeled BMDMs (Figure 7A). The drop in T2* intensity was uniform throughout the liver in axial sections of the lower (Figure 7A) and upper (supplementary Figure S5A) abdomen. The same animals were scanned again at days 13, 20 and 27 (one animal culled per time point) post-transplantation. The reduction in T2* was maintained throughout the course of the study with 42%, 56% and 49% lower T2* values at days 13, 20 and 27, respectively. Furthermore, this decrease in T2* was restricted to the liver with no differences recorded in kidney or muscle tissue (Figure 7B). T2* maps of the upper abdomen showed a similar trend with a 59%, 57%, 50% and 45% reduction in T2* time at days 6, 13, 20 and 27 (versus baseline) (supplementary Figure S5A,B). These data also suggest a slight increase in T2* relaxation time over the course of the study indicative of clearance of iron and/or cells from the liver.

**Figure 8:**
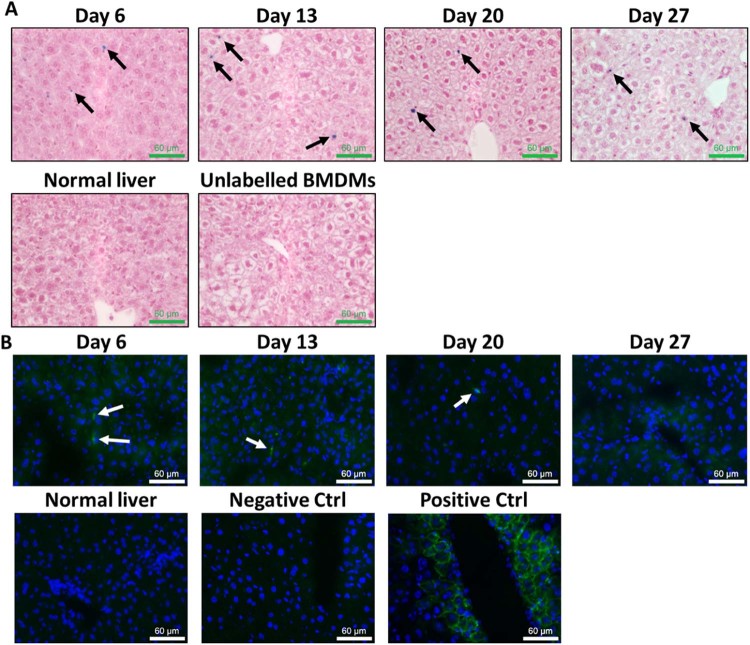
*Ex vivo* histochemical analysis confirms hepatic localization of transplanted BMDMs up to 3 weeks post-transplantation. (A) Prussian blue staining shows small punctate spots of iron (blue deposits) throughout liver parenchyma at days 6, 13, 20 and 27 post-transplantation (black arrows) against fast red counterstain, 40× magnification. (B) Immunofluorescent stains confirm hepatic localization of GFP positive cells (white arrows) in wild-type recipient frozen liver up to 20 days post-transplantation. No cells were detected at day 27, suggesting transfer of iron from donor to host cells. No GFP signal was detected in either normal C57 BL6 frozen liver or antibody-treated sections from normal frozen liver. The positive control showed strong membranous GFP staining in the livers of donor mice, confirming appropriate antigen recognition using identical conditions.

To confirm the hepatic localization, we performed Prussian blue staining and immunohistochemistry versus EGFP to detect exogenous cells in the liver. Prussian blue stains exhibited cellular punctate iron deposits throughout the liver parenchyma at all time points analyzed (Figure 8A). No iron deposits were observed in normal mouse liver or in liver that had unlabeled BMDMs transplanted. Furthermore, IHC versus EGFP on frozen liver sections showed GFP positive cells in the liver at days 6, 13 and 20. No GFP positivity was detected at day 27 suggesting possible clearance of BMDMs from the liver, similar to findings observed by Thomas *et al.*, and possible transfer of iron to host cells [11].

## Discussion

The emerging field of cell-based regenerative medicine opens new therapeutic avenues for the treatment of a range of diseases. Unlike traditional therapies using small molecules, cells have the propensity to migrate, engraft and integrate with host tissue to exert a therapeutic effect. Therefore, it is important to understand the biodistribution and pharmacokinetics of donor cells in the host tissue to monitor both the safety and efficacy of treatment. Medical imaging can be used to track cells post-transplantation, and a range of imaging modalities has been described to this end (for review, see Naumova *et al*.) [21]. One attractive approach uses MRI, a versatile, noninvasive method that offers high-resolution multidimensional imaging while providing translation of data between human and preclinical species. In this setting, contrast agents can be used to label cells *in vitro* before transplantation to provide visualization of donor cells in the host. Several iron-based contrast agents (e.g., SPIONs) have previously been used to directly label cells for cell tracking due to their high biocompatibility, high sensitivity and relatively simple labeling procedure [4], [22], [23]. However, as of 2016, three clinically approved SPIONs ferumoxide (Endorem, Feridex), ferucarbotran (Resovist, Cliavist) and ferumoxtran (Sinerem, Combidex) have all been discontinued for commercial reasons, resulting in a lack of suitable contrast agents to continue the evaluation of these molecules as cell tracking agents. Furthermore, other SPIONs that are still under manufacture such as ferumoxytol (Rienso, Feraheme) were not designed for cell labeling, so these are limited by suboptimal labeling efficiency in macrophages.

Our group has recently described a technique to generate SPIONs that harbor a functionalized dextran polymer coating that can be tailored to control iron uptake in stem cells [9]. In this work, we have investigated the effect of the particle chemistry to control the physicochemical properties of the SPION. In this way, we can identify the optimal particle characteristics required for the most efficient labeling of macrophages, a highly promising cell-based therapy for liver disease [11] currently under evaluation in phase I/II clinical trials (Macrophage therapy for Liver Cirrhosis, MATCH, Clinical Database Number 2015-000963-15). First, we demonstrate that macrophages of mouse and human origin can tolerate iron-labeling using previously available clinically approved SPIONs, resulting in negligible changes to cell health markers (Figure 1). Ferumoxide provided substantial iron uptake in cells (>5 pg Fe/cell, Figure 1C,F) whereas ferumoxytol was less efficient at labeling BMDMs (<2 pg/cell, Figure 1C). Ferumoxytol has been used for MRI-based cell tracking, but labeling concentrations of at least 500 µg/mL are required with the use of transfection agents [24]. These data confirmed that BMDMs can tolerate iron concentrations required for *in vivo* MRI detection. To improve iron-labeling efficiency, we synthesized three SPIONs with different coatings to confer a range of electrostatic charges (−11.6 mv to +19.6 mV). We observed that a positively charged variant provided the best uptake but was limited by cytotoxicity at high SPION concentrations (Figure 2A,B). By altering the molar ratios of DEAE-dextran to iron salt, we identified a SPION with a larger hydrodynamic diameter that further improved iron uptake (>10 pg Fe/cell), more so than any of the commercial variants but, importantly, reduced cytotoxicity (Figure 2D,E).

We further sought to reduce cytotoxicity by reducing the labeling time from 24 h to 4 h. This shortened protocol still provided substantial iron labeling (~5 pg Fe/cell) but vastly improved the cytotoxicity profile in both mouse (Figure 3A–C) and human macrophages (Figure 3K–M). Generally, hMDMs appeared more sensitive than BMDMs to iron labeling using the same SPION, but labeling at iron concentrations of 25 µg Fe/mL and below was demonstrated to be safe in macrophages from both species. The molecular mechanisms that underpin SPION-induced cytotoxicity are not well understood, but several mechanisms have been proposed, including induction of mitochondrial dysfunction and generation of reactive oxygen species during iron catabolism [25]. Importantly for cell tracking, the iron label was retained (>4 pg Fe/cell) for 7 days *in vitro* without any evidence of cytotoxicity (Figure 3H–J) using the shorter labeling protocol.

The ultimate aim of labeling macrophages in this study is for use in regenerative therapy. Therefore, it is imperative that they retain their function and phenotype after iron labeling. We assessed the effect of labeling BMDMs with ferumoxytol and DEAE-Dex 1:4 on the phagocytic capacity of BMDMs (Figure 4). We observed that ferumoxytol labeling caused a twofold reduction in phagocytosis whereas DEAE-Dex 1:4 labeling had no effect despite providing 10 times more iron uptake. Macrophages display a high degree of plasticity, which has led to the adoption of a simplified classification nomenclature; classical-activation (M1-like) and alternative-activation (M2-like) [26]. Because it may be important for macrophages to retain their phenotypic plasticity to exert their therapeutic effect, we investigated the effect of DEAE-Dex 1:4 labeling on BMDM phenotype via quantification of cell surface markers and gene expression analysis. The expression of CD86 (M1 marker) was increased, whereas CD206 (M2 marker) was decreased after DEAE-Dex 1:4 labeling indicating a shift toward an M1-like phenotype (Figure 5). This was further evidenced by the increased mRNA expression of M1 markers (IL-1b, IL-12b and TNFα) and associated decrease in expression of M2 markers (CD206 and Chi3L3) (Figure 6). However, this shift toward an M1-like phenotype could be reversed through the subsequent incubation of the SPION-labeled BMDMs with IL-4 as evidenced by a switch to a more M2-like gene expression profile. Taken together, these data suggest that DEAE-Dex 1:4 labeling has no effect on the phagocytic function of BMDMs but shifts phenotype toward a reversible M1-like phenotype which has been described previously [27].

Finally, we performed a transplantation study in healthy mice to test whether SPION-labeled BMDMs could be detected via MRI using the cell number and delivery route required for liver fibrosis therapy. We utilized BMDMs obtained from mice that constitutively express EGFP [16] (Figure S3, Figure S4) in BMDMs to confirm exogenous cell localisation *ex vivo*. Importantly, the liver T2* time in healthy mice was approximately 14 ms, which was consistent throughout the liver and between animals, providing a steady baseline measurement. However, 6 days after transplantation, we observed a twofold reduction in T2* time specifically in liver tissue in all mice (Figure 7A,B). This drop in T2* time was uniform throughout the liver, indicative of a homogenous distribution of BMDMs in the liver parenchyma. The reduction in T2* time was maintained at days 13, 20 and 27. We show that both iron-positive and EGFP-positive cells are present in host liver tissue (Figure 8) at days 6, 13 and 20 post-transplantation. However, no exogenous cells were detected in liver parenchyma at 27 days, suggesting clearance of these cells by unknown mechanisms, concordant with previous observations [11]. The fact that iron deposits were still visualized in liver tissue at this time and that T2* time was not yet normalized suggests that SPIONs may have transferred from transplanted cells to host cells as BMDMs are cleared from the liver. This highlights one limitation of using SPION-labeling for cell tracking: free SPIONs (e.g., SPIONs released from necrotic cells) are naturally cleared by the reticuloendothelial system, including Kupffer cells [28], thereby providing the possibility of false-positive MRI hypointense signals from host cells. It should also be noted that SPIONs are biodegradable and therefore have a finite lifetime *in vivo* becoming assimilated into the normal blood pool within weeks [29]. Thus, SPION labeling is not a reliable cell-tracking technique for long-term (>1 month) monitoring. It should also be noted that MRI cannot be used for whole-body biodistribution studies because luminal organs such as lung and stomach do not provide reliable MRI signals, whereas iron-rich organs (e.g., spleen) have a very low T2* time, vastly reducing the dynamic range of hypointensity that can be provided by SPION-labeled cells. Despite these inherent limitations, the MRI contrast provided accurate localization of transplanted BMDMs in liver for 3 weeks *in vivo*.

In conclusion, by tuning the synthetic chemistry, we have identified a novel SPION optimized for labeling macrophages of mouse and human origin without the need for transfection agents. The SPION contains a functionalized dextran polymer coating containing DEAE, which provides a positive charge for enhanced cell uptake, and fluorescein isothiocyanate, a fluorescent moiety useful for a range of *in vitro* applications. Iron labeling using DEAE-Dex 1:4 was more efficient than ferumoxytol, the only clinically approved SPION still manufactured. Furthermore, after a safe labeling protocol was determined, hepatic localisation of BMDMs could be monitored accurately up to 3 weeks post-transplantation via MRI. Further studies are now required to determine if SPION-labeled BMDMs are functionally therapeutic in the setting of liver disease. If this can be demonstrated then this clinically approved class of contrast agents may represent a useful tool to monitor safety and efficacy of macrophage therapy in patients.

## Figures and Tables

**Table I:** Summary of various SPIONs used in this current study, dextran polymers used, polymer-to-iron ratio and the resultant hydrodynamic size and surface charge.

Polymer type	Polymer-to-iron salt ratio	Hydrodynamic diameter (nm)	Surface charge (mV)	Intracellular iron content after 4 h labeling (pg) (25 µg/mL labeling)	Intracellular iron content after 24 h labeling (pg) (25 µg/mL labeling)
Dextran	4:1	36.0	−3.3	N/A	0.3
CM-dextran	4:1	34.3	−11.6	N/A	0.5
DEAE-dextran	4:1	39.7	+19.6	N/A	2.0
DEAE-dextran	1:1	47.3	+17.7	N/A	2.3
DEAE-dextran	1:4	68.0	+16.8	7.4	9.1
Ferumoxide	Unknown	115	−6.7	2.2	4.9
Ferumoxytol	Unknown	24.9	−24.2	0.1	0.9

Further information on commercial SPIONs can be found in a review by Li *et al.*[19].

## Appendix Supplementary material

Appendix S1 Supplementary methods. Appendix S1

Figure S1 Particle size distributions of (A) SPIONs synthesized with different polymers using 4:1 polymer to iron salt ratio. (B) DEAE-Dex-coated SPION synthesis using different polymer to iron salt ratios. (C) Zeta potentials of SPIONs synthesized using different dextran polymers with 4:1 polymer to iron ratio. (D) DEAE-Dex-coated SPIONs synthesized using different polymer to iron salt ratios. Figure S1

Figure S2 (A) Particle size distributions and (B) zeta potentials of the commercial ferumoxide and ferumoxytol. Figure S2

Figure S3 FACS analysis of aEGFP BMDMs shows 98% GFP positivity compared with wild-type equivalents. Bone marrow was harvested from homozygous mice expressing activated EGFP. BMDMs were differentiated according to the standard 7-day protocol. Cell preparations were incubated with antibodies for CD45, CD11b and F4/80. A DAPI stain served as a live/dead discriminator. Live single cells positive for CD45, were gated based on their expression of F4/80 and Cd11b. Positive gating was assigned based on FMO (fluorescence minus one) intensity. Figure S3

Figure S4 GFP positivity detected by immunocytochemistry in BMDMs generated from aEGFP double homozygous mice 48 h after SPION labeling. (A) Residual cell suspensions from transplantation were plated in four-well chamber slides and acclimatized in DMEM for 48 h in culture. Cells were fixed, permeabilized and blocked before incubation with anti-GFP antibodies (or isotype control). Cells were visualized by immunofluorescence using DAPI to visualise nuclei at 20× magnification. (B) Prussian blue staining confirms high and uniform iron enrichment in SPION-labeled cells 48 h after labeling in residual injectate (10× magnification). Figure S4

Figure S5 MRI detection of SPION-labeled macrophages indicates hepatic localization. (A) Axial T2* maps of the upper abdomen were generated from eight echo times in mice before (baseline, day 0) and after (days 6, 13, 20 and 27) transplantation of 1 × 10^6^ SPION-labeled BMDMs into the hepatic portal vein. False-color maps show representative T2* maps per time point. Liver tissue takes up the majority of the map (white arrows) with vasculature visible and the stomach positioned in the lower right of each axial scan. A drop in liver T2* relaxation is characterized in the maps by a switch from green (baseline) to blue (post transplantation). (B) Three ROIs were taken from liver to quantify an average mean from pixel analysis on an animal per animal basis. Consecutive MRI scans from the same mice are connected by black lines. *P* values are provided from statistical analysis where n ≥ 3, one-way analysis of variance. Figure S5
