# Supplementary material for: Functionalized superparamagnetic iron oxide nanoparticles provide highly efficient iron-labeling in macrophages for magnetic resonance–based detection in vivo
Source: Cytotherapy. 2017 Apr;19(4):555–69. doi: 10.1016/j.jcyt.2017.01.003 (PMC5357746; doi:10.1016/j.jcyt.2017.01.003)
Supplement: Appendix S1 — Supplementary methods. [file mmc1.docx]

**Supplementary Methods**

**SPION synthesis and purification**

FITC-carboxymethyl-dextran, FITC-dextran and FITC-DEAE-dextran were employed at a near 4:1 polymer to iron salt ratio [8], FITC-DEAE-dextran was also used in 1:1 and 1:4 polymer to iron salt ratios [12, 29]. For each synthesis either 0.2 (4:1), 0.05 (1:1) or 0.0125 g (1:4) of polymer was dissolved into 10 mL water and added to 0.03 g (1.1 x 10^-4^ mol) ferric chloride hexahydrate (FeCl_3_.6H_2_O) and 0.015 g (7.5 x 10^-5^ mol) ferrous chloride tetrahydrate (FeCl_2_.4H_2_O) in 15 mL water in a two necked flask fitted to a non-magnetic stirrer through an airtight connection. The flask was purged with nitrogen for 30 mins on ice and whilst stirring at 200 rpm, 1 mL ammonium hydroxide (28-30 %) was added dropwise over a period of 120 s. The mixture was then heated to 80 °C over a period of 15 mins and held at 80 °C for 1 hr. After cooling to room temperature, the reaction mixture was dialysed repeatedly using 100 KDa membrane until the particle solution reached pH 7. The particles were passed through G-100 sephadex® beads and spin-concentrated 3 times using 15 mL water containing 0.01 % sodium azide. When spin-concentrating, 1 mL of solution was left in the filter compartment between each cycle to avoid drying up the SPIONs. All particles were passed through a sterile 0.22 µm polyethersulfone syringe filter prior to cell labelling.

**Cell harvests**

Macrophages (mouse and human) were harvested by detaching cells from the surfaces of low-attachment flasks by striking the flasks by hand to encourage detachment. Cell suspensions were transferred to falcon tubes and flasks were further rinsed with PBS to capture residual cells. Cell suspensions were centrifuged (400 g, 5 mins) and resuspended in Dulbecco’s Phosphate Buffered Saline (PBS) (Sigma-Aldrich) for counting. Cells were counted using a haemocytometer (Hawksley, Lancing, UK) and 0.4 % trypan blue was used to help visualise cells and confirm high cell viability (> 90 %). For cell health assays, cells were centrifuged again and resuspended in supplemented DMEM/F:12 media (+ mCSF) for seeding into 96-well flat-bottom microplates (Corning Inc.) at 4 x 10^4^ cells/well. Cells were allowed to adhere overnight before assays were performed.

**Cell viability (ATP determination)**

Cell viability was assessed by measurement of cellular ATP content using CellTiter-Glo® Luminescent Cell Viability Assay (Promega) according to the manufacturer’s guidelines with minor modifications. Briefly, media containing SPIONs was removed to waste before cells were washed gently with PBS. Fresh supplemented media (100 µL) was added to each well before addition of ATP reaction mixture (20 µL). Microplates were placed on a shaking incubator for 60 s to lyse cells before a further 5 mins incubation in a dark environment at room temperature. Cell lysate was transferred to white 96‑well polystyrene microplates (Corning Inc. Corning, NY). Plates were read on a GloMax® 96 Microplate Luminometer (Promega) using an integration time of 1 s. Cells treated with 2 % (v/v) Triton X-100 (Sigma) served as a positive cytotoxic control.

**Cytotoxicity (LDH release determination)**

Cytotoxicity was determined using the Cytotoxicity Detection Kit based on lactate dehydrogenase leakage (LDH) (Roche) according to the manufacturer’s instructions. Briefly, media (50 µL) from cells after SPION culture were transferred to a fresh microplate. The remainder of the media was then discarded before replacement of media containing 2 % Triton X-100 (v/v) to completely lyse cells. After 5 mins incubation at room temperature, 5 µL of cell lysate was added to wells containing 45 µL of fresh media in the new microplate. The reaction was initiated by addition of 50 µL of fresh reaction mixture (made according the manufacturer’s instructions) to each well. Plates were incubated in dark conditions for 30 mins at room temperature to allow the color change to develop. Microplates were read on a FLUOstar Omega microplate reader (BMG Labtech) using colorimetric absorbance at 492 nm.

**Prussian blue staining**

Cellular iron content was visualised using the Prussian blue stain. BMDMs were seeded in Lab-Tek II 4-well chamber slides at 2x10^5^ cells/well and allowed to adhere overnight. BMDMs were incubated with media containing SPIONs (0-100 µg Fe/mL) for up to 24 hrs. For staining, media were aspirated and washed gently with PBS. Chambers were removed and slides immersed in distilled H_2_0 for 5 mins before incubation in acidified potassium ferrocyanide (10 % HCl, v/v.; 5 % K_4_Fe(CN)_6_, w/v) solution for 20 mins. Slides were washed and counterstained with neutral red for 2 mins before washing and mounting in aqueous fixative. Slides were images on a light microscope. For Prussian blue staining in liver tissue, 4 µm sections were cut from formalin-fixed paraffin-embedded blocks and collected on Superfrost™ Plus slides (Thermo Fisher). Sections were cleared in xylene and rehydrated in decreasing concentrations of ethanol. Staining was identical to cells except the counterstain was nuclear fast red (10 mins). Sections were dehydrated and mounted in Pertex before imaging on a light microscope (Nikon Eclipse E600).

**Gene expression analysis**

The expression of a number of mRNAs chosen for their role in macrophage polarisation were quantified using quantitative PCR (qPCR). BMDMs were prepared and incubated with SPIONS as described previously for 24 hrs. RNA was isolated from BMDMs using a phenol-chloroform extraction method. Purified RNA (3 µg) was DNase treated and reverse transcribed to cDNA through incubation with random hexamers, deoxynucleotide solution (dNTP), Dithiothretol (DTT) and reverse transcriptase (SuperScrpt III, ThermoFisher). cDNA was diluted 1 in 10 and used for SYBR Green based qPCR (SYBR Green JumpStart Taq ReadyMix, Sigma) using the following conditions: 95 °C, 5 mins followed by 40 cycles of 95 °C for 10 s then 60 °C for 30 s. cDNA was incubated with the following primers; IL-1b, IL-12b, IL-10, TNFα, CD206 (MRC), Arg-1, Chi3L3 (QuantiTect Primer Assay, QIAGEN). Each sample was measured in triplicate and normalised to GAPDH.

**Flow cytometry**

In order to assess the phagocytic ability of BMDMs incubated with SPIONs, BMDMs were incubated with fluorescently-labelled *S. cerevisiae* (Zymosan A *S. cerevisiae* BioParticles, Texas Red conjugated, ThermoFisher Scientific). BMDMs (1 x 10^6^ cells) were treated as described previously then media aspirated and replaced with media containing fluorescently-labelled *S. cerevisiae* (~10 yeast particles per BMDM). BMDMs were then incubated with the *S. cerevisiae* for 2 hrs before aspiration of the media and subsequent washes with PBS. BMDMs were then removed from the plate using trypsin, centrifuged, and resuspended in PBS. Extracellular Texas Red was quenched by incubation with 0.4 % Trypan Blue for 30 min before 2 wash steps. Cells were then resuspended in PBS (500 µL) and Texas Red was detected using flow cytometry (Becton Dickinson FACSCalibur). Parameters were set using a sample containing BMDMs alone and with a sample containing *S cerevisiae* only.

Flow cytometry was also used to assess the cell surface markers CD86 and CD206 on BMDMs. Briefly, BMDMs were incubated with SPIONs for 24 hrs. Media containing SPIONS were aspirated and cells washed with PBS and trypsinised. Cells were centrifuged and washed before resuspension in PBS (100 µL). BMDMs were incubated with antibodies against murine CD86 and CD206 (APC anti-mouse CD86 and PerCP/Cy5.5 anti-mouse CD206 respectively, Biolegend) for 15 min. Cells were then washed twice before resuspension in PBS (500 µL). Cells were then detected using flow cytometry. Untreated BMDMs and BMDMs incubated with only CD86 or CD206 antibodies were used to set parameters. For GFP analysis, BMDMs from aEGFP mice were harvested and resuspended in PBS (1% FCS) and filtered through a 70 μm filter. BMDM suspensions were stained with 0.25 μg/mL CD11b PE (BD Pharmingen Clone: M1/70), 0.25 μg/mL F4/80 PE Cy7 (BioLegend Clone: BM8) and 1 μg/mL CD45 APC (eBioscience Clone: 30-F11) for 15 mins at 4 °C. Cells were washed with 1 mL FACS wash and resuspended in 300 μL 0.1 μg/mL DAPI FACS wash. FMOs for each color were prepared in the same manner. 1 drop of UltraComp eBeads (eBioscience) were stained in 200 μL FACS wash for 10 mins at 4 °C using identical concentrations of each antibody then washed in 1 mL FACS wash and centrifuged as above. FMOs were used to set analysis gates. GFP cells are: single, live, CD45+, F4/80+, CD11b+.
