# Supplementary figures and images for: Functionalized superparamagnetic iron oxide nanoparticles provide highly efficient iron-labeling in macrophages for magnetic resonance–based detection in vivo
Source: Cytotherapy. 2017 Apr;19(4):555–69. doi: 10.1016/j.jcyt.2017.01.003 (PMC5357746; doi:10.1016/j.jcyt.2017.01.003)

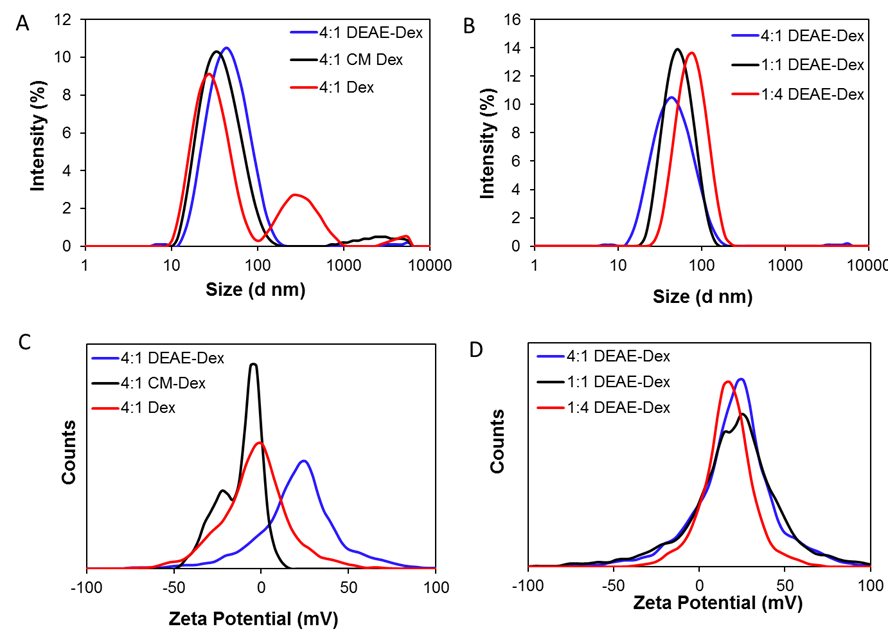

Supplement: Figure S1 — Particle size distributions of (A) SPIONs synthesized with different polymers using 4:1 polymer to iron salt ratio. (B) DEAE-Dex-coated SPION synthesis using different polymer to iron salt ratios. (C) Zeta potentials of SPIONs synthesized using different dextran polymers with 4:1 polymer to iron ratio. (D) DEAE-Dex-coated SPIONs synthesized using different polymer to iron salt ratios. [file mmc2.zip › mmc2.tif]

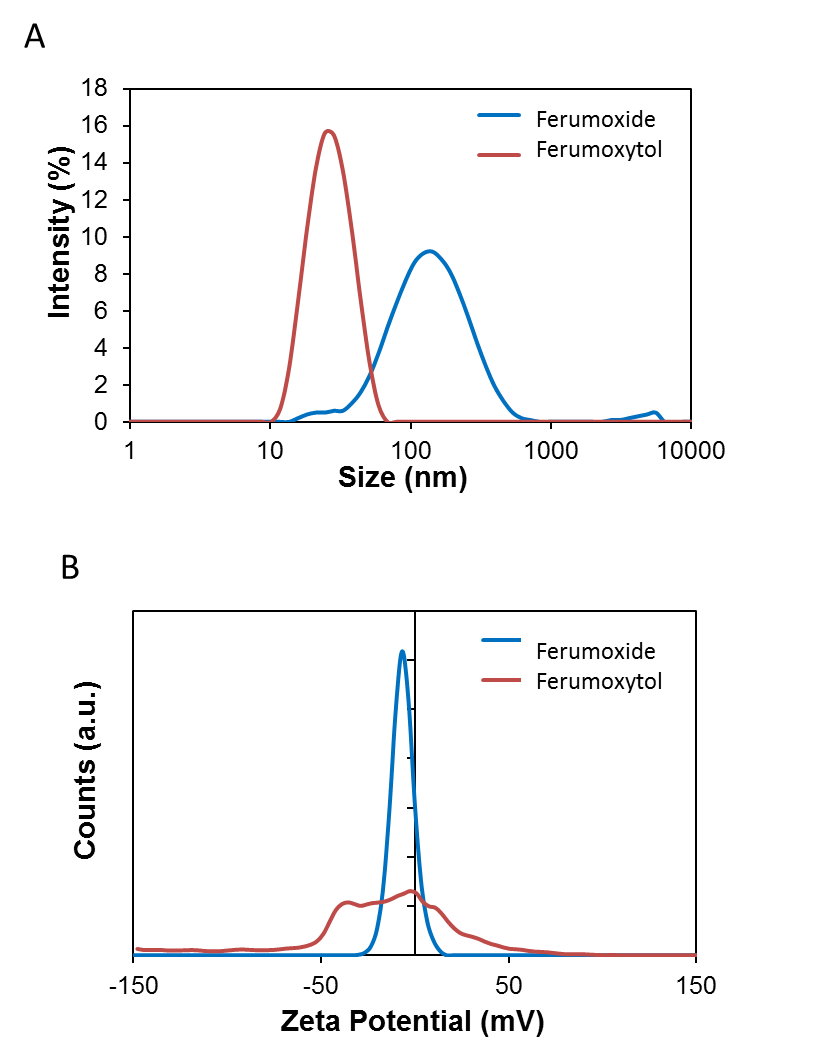

Supplement: Figure S2 — (A) Particle size distributions and (B) zeta potentials of the commercial ferumoxide and ferumoxytol. [file mmc3.zip › mmc3.tif]

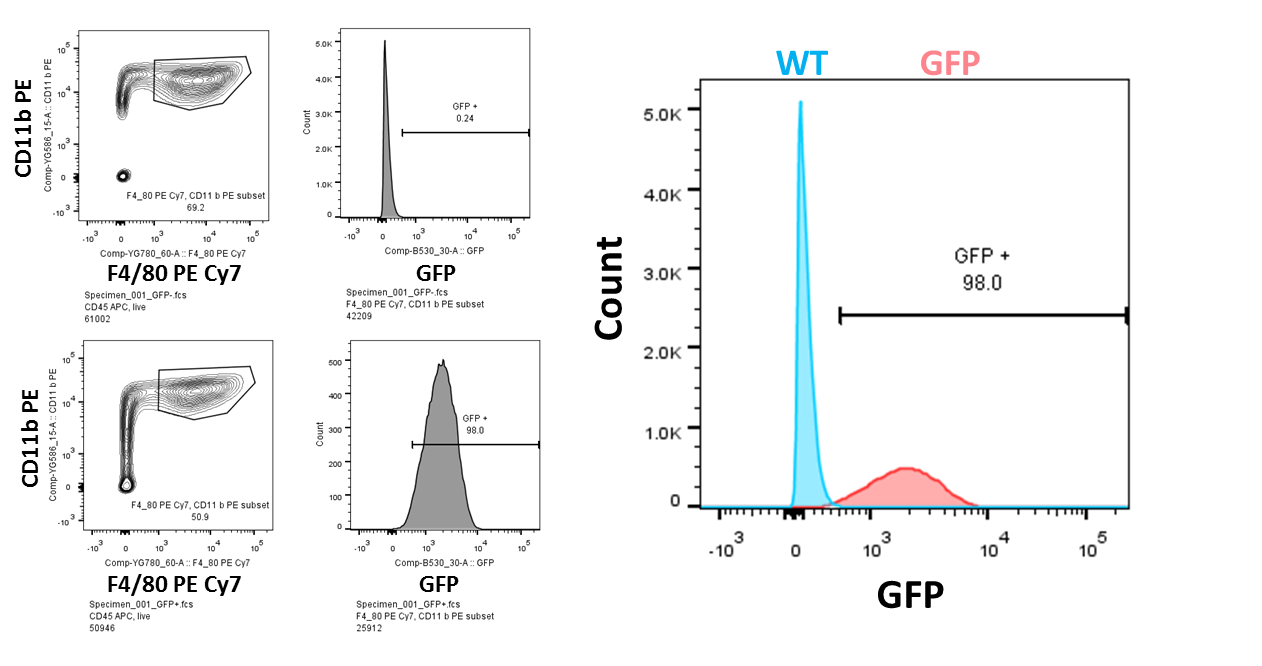

Supplement: Figure S3 — FACS analysis of aEGFP BMDMs shows 98% GFP positivity compared with wild-type equivalents. Bone marrow was harvested from homozygous mice expressing activated EGFP. BMDMs were differentiated according to the standard 7-day protocol. Cell preparations were incubated with antibodies for CD45, CD11b and F4/80. A DAPI stain served as a live/dead discriminator. Live single cells positive for CD45, were gated based on their expression of F4/80 and Cd11b. Positive gating was assigned based on FMO (fluorescence minus one) intensity. [file mmc4.zip › mmc4.tif]

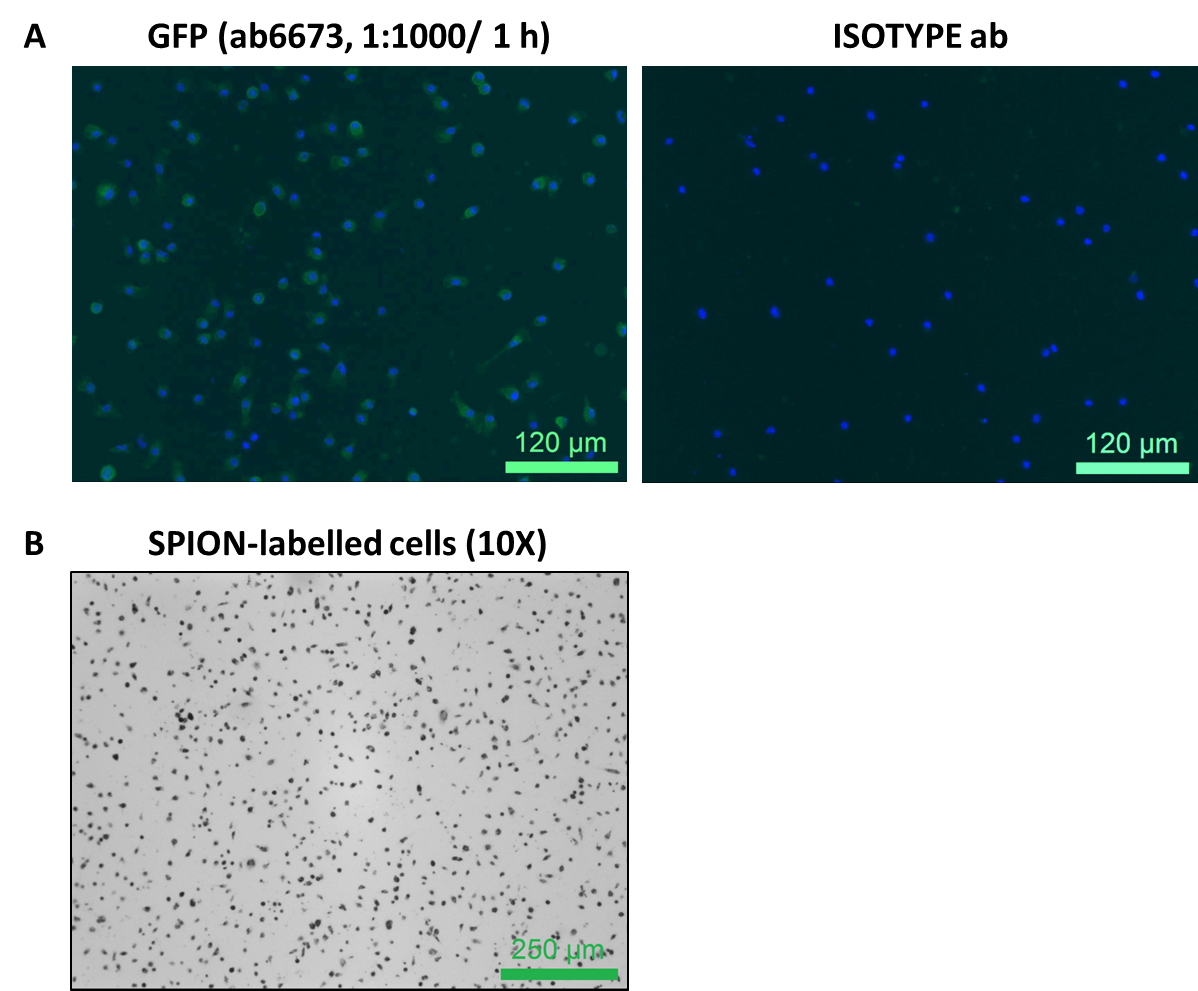

Supplement: Figure S4 — GFP positivity detected by immunocytochemistry in BMDMs generated from aEGFP double homozygous mice 48 h after SPION labeling. (A) Residual cell suspensions from transplantation were plated in four-well chamber slides and acclimatized in DMEM for 48 h in culture. Cells were fixed, permeabilized and blocked before incubation with anti-GFP antibodies (or isotype control). Cells were visualized by immunofluorescence using DAPI to visualise nuclei at 20× magnification. (B) Prussian blue staining confirms high and uniform iron enrichment in SPION-labeled cells 48 h after labeling in residual injectate (10× magnification). [file mmc5.zip › mmc5.tif]

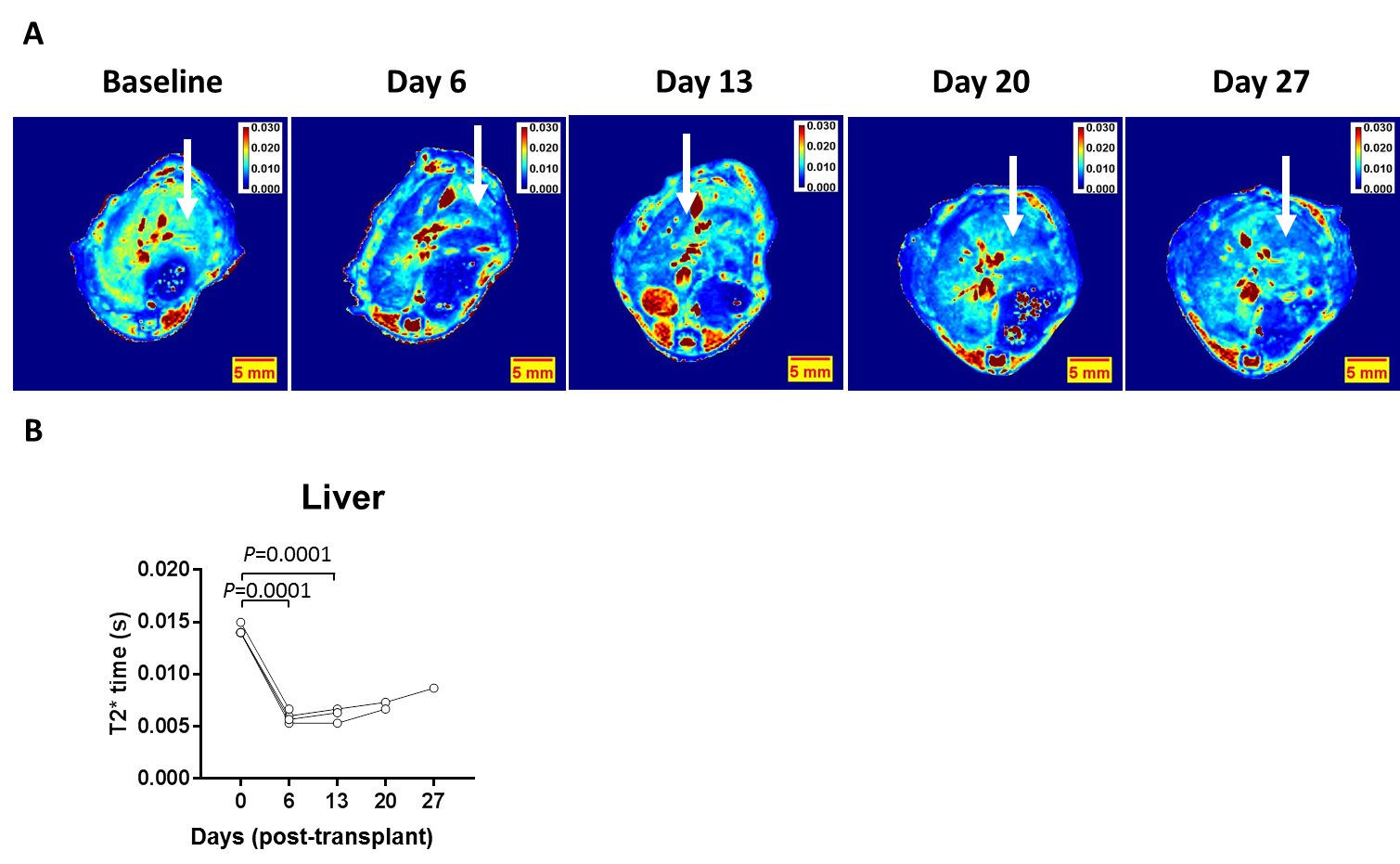

Supplement: Figure S5 — MRI detection of SPION-labeled macrophages indicates hepatic localization. (A) Axial T2* maps of the upper abdomen were generated from eight echo times in mice before (baseline, day 0) and after (days 6, 13, 20 and 27) transplantation of 1 × 10^6^ SPION-labeled BMDMs into the hepatic portal vein. False-color maps show representative T2* maps per time point. Liver tissue takes up the majority of the map (white arrows) with vasculature visible and the stomach positioned in the lower right of each axial scan. A drop in liver T2* relaxation is characterized in the maps by a switch from green (baseline) to blue (post transplantation). (B) Three ROIs were taken from liver to quantify an average mean from pixel analysis on an animal per animal basis. Consecutive MRI scans from the same mice are connected by black lines. P values are provided from statistical analysis where n ≥ 3, one-way analysis of variance. [file mmc6.zip › mmc6.tif]
